# Personal hair dye use and the risk of bladder cancer: a case–control study from The Netherlands

**DOI:** 10.1007/s10552-012-9982-1

**Published:** 2012-05-13

**Authors:** Martine M. Ros, Manuela Gago-Dominguez, Katja K. H. Aben, H. Bas Bueno-de-Mesquita, Ellen Kampman, Sita H. Vermeulen, Lambertus A. Kiemeney

**Affiliations:** 1 Department of Epidemiology, Biostatistics and HTA, Radboud University Medical Centre, P.O. Box 9101, 6500 HB Nijmegen, The Netherlands; 2 National Institute for Public Health and the Environment (RIVM), Bilthoven, The Netherlands; 3 University Hospital Santiago de Compostela, Santiago de Compostela, Spain; 4 Comprehensive Cancer Centre the Netherlands, Utrecht, The Netherlands; 5 Department of Gastroenterology and Hepatology, University Medical Centre, Utrecht, The Netherlands; 6 Division of Human Nutrition, Wageningen University, Wageningen, The Netherlands; 7 Department of Urology, Radboud University Medical Centre, Nijmegen, The Netherlands

**Keywords:** Bladder cancer, Hair dyes, Permanent hair dye, Temporary hair dye, Case–control study

## Abstract

**Background:**

Several studies have suggested an increased risk of bladder cancer among hairdressers, who are occupationally exposed to hair dyes. There has also been concern about a possible increased risk of bladder cancer among users of hair dyes. However, the association between personal hair dye use and bladder cancer risk remains inconclusive.

**Objective:**

In this study, we examined associations between personal use of permanent and temporary hair dyes and bladder cancer risk in a population-based case–control study involving 1,385 cases (*n* = 246 women) and 4,754 controls (*n* = 2,587 women).

**Methods:**

Participants filled out a questionnaire with regard to history of personal hair dye use and risk factors for bladder cancer. Unconditional logistic regression was used to calculate odds ratios (OR) and 95 % confidence intervals (CI), adjusted for age, smoking status, duration of smoking and intensity of smoking.

**Results:**

Analyses were restricted to women as less than 5 % of all men in the study ever used hair dyes. About 50 % of the women ever used hair dyes. Use of temporary hair dyes (OR, 0.77; 95 % CI, 0.58–1.02) or use of permanent hair dyes (OR, 0.87; 95 % CI, 0.65–1.18) was not associated with bladder cancer risk. No clear association between hair dyes and bladder cancer risk was found when dye use was defined by type, duration or frequency of use, dye color, or extent of use. Also, results were similar for aggressive- and non-aggressive bladder cancer. Age, educational level, and smoking status did not modify the association between hair dye use and bladder cancer risk.

**Conclusions:**

The present study does not support an association between personal hair dye use and bladder cancer risk. Also, various types of hair dye, intensity of exposure to hair dyes or dye color do not appear to be important factors for bladder cancer development.

## Introduction

Occupational exposure to hair dyes has been associated with an increased risk of bladder cancer [1, 2]. The International Agency for Research on Cancer (IARC) concludes that hairdressers and barbers are “probably” at greater risk of bladder cancer because of their exposure to hair dyes [3]. This has raised concerns that personal use of hair dyes may also increase the risk of bladder cancer [4]. Personal hair dyes are widely used. It has been estimated that over one-third of women above the age of 18 and over 10 % of men above the age of 40 in Europe and North America use some type of hair dye [5].

Small amounts of 4-aminobiphenyl (4-ABP), a recognized urinary bladder carcinogen which is banned since the mid-1950s from the US market and since 1998 from the European market [6], were identified in commercial hair dyes yet. The amount of 4-ABP found in hair dyes varies, and frequent use of hair dyes may result in considerable exposure to 4-ABP over time [7]. These and other aromatic amines and related nitro-compounds in hair dyes are known to be mutagenic in vitro [3, 8] and carcinogenic in animal models [9]. Small amounts of these substances are absorbed through the skin during normal use [10]. Oxidation of aromatic amines to *N*-hydroxyarylamine is thought to be required for carcinogenic potency [11, 12]. Hair dyes comprise a complex group where oxidative (permanent) dyes differ in composition compared to non-oxidative (semi-permanent and temporary) dyes, although carcinogenic agents have been found in each type of hair dye. Permanent dyes consist of primary intermediates (e.g., *p*-phenylenediamines, *p*-aminophenols) and couplers (e.g., *m*-aminophenols, *m*-hydroxyphenols) that, in the presence of peroxide, form the dye by a chemical reaction. Non-oxidative hair dyes include colored compounds that stain hair directly. Semi-permanent hair dyes resist several shampooings, while temporary dyes wash out after one shampooing [4, 13].

The possible association between personal hair dye use and bladder cancer risk has been examined in several epidemiological studies [14–24]. Overall, these studies did not show an association with bladder cancer risk. However, most studies on bladder cancer risk associated with personal hair dye use suffer from methodological difficulties. Several studies did not collect information on type and color of hair dyes. This may have led to attenuation of the risk estimate because permanent dyes (particularly dark dyes) are considered to pose a greater risk than other types of hair coloring products. Small sample sizes in most studies limited the statistical power to detect an effect if one exists. In addition, some studies [14, 15, 18] examined only death rates of bladder cancer instead of incidence rates, which is inappropriate since most bladder cancers (approximately 75 %) involve only the superficial layers of the bladder and can be cured either with chemotherapy or surgical removal of the bladder**.**

Despite the majority of studies reporting null associations, personal hair dye use has led to discussions about its impact on human health. A case–control study from California found an increased risk of bladder cancer in long-term (15 years and more) users of permanent hair dyes among women [22], which was more pronounced among exclusive users and women with the *N*-acetyltransferase-2 (NAT2) slow acetylator phenotype. Also, a recent study from New England found that women who used permanent dyes and had a college degree had an increased risk of bladder cancer and that the risk was more pronounced among exclusive users of permanent hair dyes who also had the NAT2 slow acetylation phenotype [24].

Because of the widespread use of hair dyes, a small increase in risk of bladder cancer may have a large public health impact. The aim of the present study was to investigate associations between personal use of permanent and temporary hair dyes and bladder cancer risk in a case–control study from the Netherlands.

## Methods

### Study population

Patients diagnosed with bladder cancer between 1975 and 2009 under the age of 75 years registered by the population-based cancer registry in the eastern part of the Netherlands were invited to participate in a study on genes and environment as potential risk factors for bladder cancer (The Nijmegen Bladder Cancer Study, NBCS). Patients were requested to fill out a detailed questionnaire on lifestyle and medical factors and to donate a blood sample for DNA isolation. The response rate for cases was 62 %. All cases were histologically confirmed. For the current analysis, only cases with urothelial cell carcinoma (morphology ICD-O-3 codes 8120 and 8130) were included (*n* = 1,501). Data on tumor stage and grade were obtained through the cancer registry. According to Kiemeney et al. [25], we classified urothelial cell carcinomas with regard to risk of progression into aggressive and non-aggressive tumors. Patients with high-risk of progression (aggressive UCC) were defined as TNM stage Tis or T1 and higher or WHO 1973 differentiation grade 3 or WHO/ISUP 2004 high grade. Patients with low-risk of progression (non-aggressive UCC) were defined as having TNM stage Ta in combination with WHO 1973 differentiation grade 1 or 2 or WHO/ISUP 2004 low grade.

Controls were recruited for the Nijmegen Biomedical Study (NBS), a population-based survey conducted in 2002 by the Department of Epidemiology and Biostatistics in collaboration with the Department of Clinical Chemistry of the Radboud University Medical Centre. 21,756 age- and sex-stratified randomly selected inhabitants of the municipality of Nijmegen, The Netherlands, received an invitation to fill out a postal questionnaire on, for example, lifestyle and medical history, and to donate two tubes of blood. The response to the questionnaire was 43 % (*n* = 9,350). The NBS participants who gave consent for further research and were still alive in 2008 were contacted again with an invitation to fill out an additional detailed life-style questionnaire that also contained questions on personal hair dye use. In total, 5,613 (60 %) participants completed this questionnaire. Control participants who had a prior history of cancer (except non-melanoma skin cancer) at the time of recruitment were excluded (*n* = 303). A detailed description of the design and study population has been reported previously [26, 27].

The study protocols of the NBCS study and the NBS were approved by the Institutional Review Board of the Radboud University Medical Centre, and all participants gave written informed consent.

### Assessment of personal hair dye use

Participants were asked about ever use of temporary and permanent hair dyes separately. In addition, information on duration of use (years), frequency of use (times per year), extent of use (part or entire scalp), and color of the most frequently used dye was collected for each type of hair dye product used. Specific colors were classified as brown, black, blond, red, and other colors. Permanent hair dye was defined as a hair color that does not wash out (but grows out) and temporary dye as a hair color that washes out over time. Participants with missing data on hair dye use (20 cases and 97 controls) and smoking variables were excluded (96 cases and 459 controls).

### Statistical analysis

Unconditional logistic regression models were used to estimate odds ratios (OR) and 95 % confidence intervals (95 % CI) for the association between type of personal hair dye use (temporary or permanent dyes, both temporary and permanent dyes, exclusive temporary or exclusive permanent dyes) and bladder cancer. All analyses were adjusted for age at time of completing the questionnaire (continuous), smoking status (never, ever), duration of smoking (in years), and intensity of smoking (cigarettes/day). Participants who never smoked during their life were defined as never smokers. If participants quitted smoking cigarettes, cigars, or pipe before completing the questionnaire, we defined them as former smokers. Current smokers were defined as participants who smoked cigarettes, cigars, or pipe at the time of completing the questionnaire. It is likely that some former smokers returned to smoking and that some current smokers stopped smoking just after being diagnosed with bladder cancer. Since the smoking status could have changed, we classified participants into never and ever (former and current) smokers. Because additional adjustment for height, weight, occurrence of bladder cancer in first-degree relatives and educational level (primary school, secondary school, technical, and professional school, and university degree) did not change the β estimate by more than 10 %, these factors were not included in the final analyses. Risks of bladder cancer were also evaluated by duration (≤10 or >10 years), frequency (≤5 times or >5 times a year, and ≤40 times or >40 times during lifetime), color (brown, black, blond, red, and other colors), and extent of use (part or entire scalp). Separate analyses were performed for non-aggressive and aggressive urothelial bladder cancer. Additional stratified analyses were performed on age (≤65 and >65 years of age), educational level (as a proxy for quality of answers to the questions), and smoking status (never, ever). In order to increase power, we classified educational level into no college degree (primary and secondary school) and at least a college degree (technical and professional school, and university degree). Statistical interaction on a multiplicative scale was tested by introducing a product term between hair dye use and age, educational level, or smoking status. The reference group for analyses of temporary hair dyes consisted of subjects who had never used temporary hair dyes. The reference group for analyses of permanent hair dyes consisted of subjects who had never used permanent hair dyes. An additional analysis was conducted to evaluate whether the use of another reference category (never used any type of hair dye) would yield different results. In the current study, hair dye use appeared to be rare among men: less than 5 % of both male cases and controls had ever used hair dyes. Therefore, analyses were restricted to women. The study sample size of 246 female cases and 2,587 controls was estimated to provide 80 % power at the significance level of 5 % to detect odds ratios of <0.68 or >1.25 in the category of hair dye users as compared to never users. Naturally, for the subgroup analyses, the power to detect associations was smaller. All statistical analyses were performed in SAS (SAS system for Windows, version 9.2, SAS institute, Cary, NC).

## Results

The present study included 1,385 bladder cancer cases (1,139 men and 246 women) and 4,754 controls (2,167 men and 2,587 women). On average, cases were much older, more likely to report bladder cancer in the first-degree family, had a lower level of education and were more likely to be former or current smokers than the controls. Among former and current smokers, cases had smoked for a longer period and smoked more cigarettes per day than controls. Temporary and permanent hair dyes were used more frequently by controls than by cases (Table 1). Female users of hair dyes were somewhat younger, reported more frequently a higher educational level and the percentage of users that reported to be former or current smoker was slightly higher compared to non-users (Table 2).

**Table 1:** Description of the study population

	Women		Men	
	Cases (*n* = 246)	Controls (*n* = 2,587)	Cases (*n* = 1,139)	Controls (*n* = 2,167)
Age at completion of the questionnaire (years)	66.3 ± 10.5	54.4 ± 17.0	67.7 ± 9.0	59.3 ± 16.3
Height (cm)	165.0 ± 9.7	167.4 ± 6.7	177.4 ± 8.8	178.9 ± 7.2
Weight (kg)	68.5 ± 10.6	69.2 ± 11.9	81.7 ± 12.3	81.2 ± 12.2
Bladder cancer in first-degree relative (%)	8	2	6	2
Smoking status				
Never smokers (%)	27	48	8	29
Former smokers (%)	51	38	69	55
Number of cigarettes (cig/day)	12.6 ± 7.5	10.8 ± 7.7	16.0 ± 8.5	14.1 ± 8.8
Smoking duration (y)	26.9 ± 13.8	17.7 ± 12.8	29.6 ± 13.7	22.7 ± 13.8
Age at start smoking (y)	19.6 ± 6.4	18.1 ± 5.4	17.2 ± 4.3	17.1 ± 3.8
Current smokers (%)	22	14	23	16
Number of cigarettes (cig/day)	14.3 ± 6.1	13.0 ± 7.9	15.7 ± 6.9	14.3 ± 7.7
Smoking duration (y)	40.6 ± 11.1	30.3 ± 14.2	43.6 ± 13.3	34.0 ± 15.9
Age at start smoking (y)	18.8 ± 5.9	17.4 ± 5.2	17.2 ± 4.7	17.2 ± 4.2
Educational level (%)				
Primary school	21	7	14	5
Technical/professional school	48	28	40	26
Secondary school	16	25	21	25
University degree	12	40	22	44
Not specified	3	–	3	–
Temporary hair dye use (*n*, %)^a^	105 (42)	1,393 (54)	21 (2)	105 (5)
Permanent hair dye use (*n*, %)^a^	120 (49)	1,593 (62)	16 (2)	91 (4)

Mean ± SD, unless otherwise stated

^a^Permanent dye is defined as a hair color that does not wash out (but grows out), temporary dye as a hair color that washes out over time

**Table 2:** Baseline characteristics of 2,587 controls by hair dye use in women

	Temporary dyes^a^		Permanent dyes^a^	
	Non-users (*n* = 1,194)	Users (*n* = 1,393)	Non-users (*n* = 994)	Users (*n* = 1,593)
Age at completion of the questionnaire (years)	57.1 ± 17.6	52.0 ± 16.1	60.5 ± 19.0	50.5 ± 14.3
Height (cm)	166.8 ± 6.8	167.8 ± 6.6	166.4 ± 7.0	168.0 ± 6.4
Weight (kg)	69.3 ± 12.0	69.1 ± 11.8	68.9 ± 11.6	69.4 ± 12.2
Bladder cancer in first-degree relative (%)	2	2	2	2
Smoking status				
Never smokers (%)	51	45	57	43
Former smokers (%)	36	40	33	41
Number of cigarettes (cig/day)	13.0 ± 7.8	13.0 ± 7.9	14.6 ± 6.6	14.0 ± 5.8
Smoking duration (y)	34.8 ± 16.1	28.5 ± 12.9	43.5 ± 12.2	38.4 ± 9.9
Age at start smoking (y)	18.8 ± 7.1	16.9 ± 4.2	19.0 ± 4.5	18.6 ± 6.8
Current smokers (%)	13	15	10	16
Number of cigarettes (cig/day)	12.8 ± 8.0	13.2 ± 7.7	13.7 ± 5.8	14.8 ± 6.5
Smoking duration (y)	30.9 ± 15.9	29.9 ± 13.1	41.6 ± 12.9	39.5 ± 8.9
Age at start smoking (y)	17.7 ± 5.3	17.3 ± 5.2	19.2 ± 7.4	18.3 ± 3.7
Educational level (%)				
Primary school	8	6	9	6
Technical/professional school	30	25	31	25
Secondary school	23	27	22	27
University degree	39	42	38	42

Mean ± SD, unless otherwise stated

^a^Permanent dye is defined as a hair color that does not wash out (but grows out), temporary dye as a hair color that washes out over time

The risks of bladder cancer by personal hair dye use in women are presented in Tables 3 and 4. Compared to women who never used temporary hair dyes, the OR for temporary hair dye use was 0.77 (95 % CI, 0.58–1.02) (Table 3). Use of permanent dyes was not associated with bladder cancer risk either (OR, 0.87; 95 % CI, 0.65–1.18) (Table 3). Among women using both types of personal hair dye (permanent and temporary), the OR was 0.72 (95 % CI, 0.27–1.26) compared to women who never used any type of hair dye (Table 4). Compared to women who never used any type of hair dye, the ORs for exclusive use of temporary hair dyes or permanent hair dyes were 0.71 (95 % CI, 0.47–1.09) and 0.84 (95 % CI, 0.57–1.26), respectively (Table 4). No clear association was found between hair dyes and bladder cancer risk when dye use was defined by duration of use, frequency of use (number of times a year or times during lifetime), dye color, or extent of use (entire hair or only part of hair). We found no differences in risks of aggressive- and non-aggressive bladder cancer (Tables 3 and 4). When analyses were stratified by age, educational level, or smoking status, risk estimates were not different over the strata (Table 5).

**Table 3:** Risk of bladder cancer according to duration, frequency, and color of use of hair dyes in women

	Temporary dyes^a^		Permanent dyes^a^	
	Ca/Co	OR (95 % CI)^b^	Ca/Co	OR (95 % CI)^b^
Never used hair dyes^c^	141/1,194	1.00	126/994	1.00
Used hair dyes	105/1,393	0.77 (0.58–1.02)	120/1,593	0.87 (0.65–1.18)
Duration of use				
Never used hair dyes	141/1,194	1.00	126/994	1.00
Used hair dyes ≤ 10 years	69/1,147	0.69 (0.50–0.94)	58/1,032	0.76 (0.53–1.09)
Used hair dyes > 10 years	36/246	1.02 (0.67–1.53)	62/561	1.01 (0.71–1.44)
Number of times per year^d^				
Never used hair dyes	141/1,194	1.00	126/994	1.00
Used hair dyes ≤ 5 times a year	63/884	0.88 (0.63–1.23)	61/911	0.91 (0.63–1.31)
Used hair dyes > 5 times a year	40/404	0.80 (0.55–1.17)	57/635	0.90 (0.63–1.28)
Lifetime use (total number of times)^e^				
Never used hair dyes	141/1,194	1.00	126/994	1.00
Used hair dye ≤ 40 times	50/852	0.77 (0.54–1.11)	42/765	0.84 (0.56–1.26)
Used hair dye > 40 times	47/368	0.94 (0.65–1.35)	73/733	0.94 (0.68–1.32)
Dye entire or part of hair				
Never used hair dyes	141/1,194	1.00	26/994	1.00
Dye entire hair	98/1,201	0.85 (0.64–1.13)	104/1,257	0.92 (0.67–1.25)
Dye part of hair	6/138	0.45 (0.19–1.06)	17/364	0.59 (0.34–1.05)
Color				
Never used hair dyes	141/1,194	1.00	126/994	1.00
Blond	36/379	0.81 (0.54–1.21)	57/757	0.84 (0.58–1.22)
Brown	51/553	0.93 (0.65–1.32)	54/612	0.97 (0.67–1.41)
Black	0/64	–^f^	1/85	–^f^
Red and other colors	27/539	0.69 (0.43–1.09)	30/515	0.72 (0.45–1.16)
Prognostic subtype of bladder cancer				
Aggressive bladder cancer^g,i^				
Never used hair dyes	43/1,194	1.00	43/994	1.00
Used hair dyes	29/1,393	0.75 (0.46–1.23)	29/1,593	0.66 (0.39–1.13)
Non-aggressive bladder cancer^h,i^				
Never used hair dyes	56/1,194	1.00	56/994	1.00
Used hair dyes	50/1,393	0.89 (0.59–1.32)	50/1,593	0.74 (0.48–1.14)

^a^Permanent dye is defined as a hair color that does not wash out (but grows out), temporary dye as a hair color that washes out over time

^b^Odds ratios were adjusted for age at time of completing the questionnaire, smoking status, smoking duration, and smoking intensity using logistic regression

^c^Never users of hair dye are the referent category for each model. The reference group for analyses of temporary hair dyes consisted of subjects who had never used temporary hair dyes. The reference group for analyses of permanent hair dyes consisted of subjects who had never used permanent hair dyes

^d^For 107 participants (2 cases and 105 controls) using temporary dyes and for 49 participants (2 cases and 47 controls) using permanent dyes, information is lacking on number of times per year

^e^For 181 participants (8 cases and 173 controls) using temporary dyes and for 100 participants (5 cases and 95 controls) using permanent dyes, information is lacking on lifetime use

^f^Too few cases for reliable results

^g^The category “aggressive urothelial cell carcinomas” includes TNM stage Tis or T1 and higher or WHO 1973 differentiation grade 3 or WHO/ISUP 2004 high grade

^h^The category “non-aggressive urothelial cell carcinomas” includes TNM stage Ta grade in combination with WHO 1973 differentiation grade 1 or 2 or WHO/ISUP 2004 low grade

^i^68 cases cannot be classified as aggressive or non-aggressive urothelial cell carcinomas, because of lacking information on stage and/or grade

**Table 4:** Risk of bladder cancer among women using temporary and permanent dyes

	Both temporary and permanent dyes^a^		Exclusive temporary dyes^a^		Exclusive permanent dyes^a^	
	Ca/Co	OR (95 % CI)^b^	Ca/Co	OR (95 % CI)^b^	Ca/Co	OR (95 % CI)^b^
Never used hair dyes^c^	88/579	1.00	88/579	1.00	88/579	1.00
Used hair dyes	67/978	0.72 (0.27–1.26)	38/415	0.71 (0.47–1.09)	53/615	0.84 (0.57–1.26)
Duration of use^d^						
Never used hair dyes	88/579	1.00	88/579	1.00	88/579	1.00
Used hair dyes ≤ 10 years	35/558	0.71 (0.44–1.14)	26/276	0.76 (0.47–1.24)	18/301	0.58 (0.33–1.02)
Used hair dyes > 10 years	32/409	0.62 (0.39–1.00)	12/112	0.65 (0.34–1.26)	34/301	0.91 (0.57–1.46)
Number of times per year^e^						
Never used hair dyes	88/579	1.00	88/579	1.00	88/579	1.00
Used hair dyes ≤ 5 times a year	15/260	0.79 (0.42–1.50)	35/372	0.70 (0.45–1.09)	45/529	0.76 (0.49–1.17)
Used hair dyes > 5 times a year	52/697	0.67 (0.44–1.01)	3/16	–^g^	7/73	0.78 (0.33–1.83)
Lifetime use (total number of times)^f^						
Never used hair dyes	88/579	1.00	88/579	1.00	88/579	1.00
Used hair dye ≤ 40 times	19/369	0.79 (0.43–1.44)	21/263	0.72 (0.42–1.22)	12/253	0.52 (0.27–1.03)
Used hair dye > 40 times	46/560	0.65 (0.43–1.00)	15/102	0.75 (0.41–1.39)	39/336	0.86 (0.55–1.35)
Dye entire or part of hair						
Never used hair dyes	88/579	1.00	88/579	1.00	88/579	1.00
Dye entire hair	57/747	0.70 (0.46–1.06)	37/347	0.80 (0.52–1.23)	45/451	0.86 (0.56–1.31)
Dye part of hair	4/55	–^g^	1/50	–^g^	7/178	–^g^
Color						
Never used hair dyes	88/579	1.00	88/579	1.00	88/579	1.00
Blond	22/231	0.73 (0.43–1.25)	14/118	0.71 (0.38–1.32)	26/349	0.69 (0.41–1.15)
Brown	29/308	0.90 (0.56–1.47)	21/161	0.95 (0.55–1.61)	20/199	0.86 (0.50–1.50)
Black	0/29	–^g^	0/15	–^g^	1/28	–^g^
Red and other colors	17/281	0.58 (0.31–1.08)	6/129	0.48 (0.19–1.17)	10/169	0.54 (0.25–1.14)
Prognostic subtype of bladder cancer						
Aggressive bladder cancer^h, j^						
Never used hair dyes	32/579	1.00	32/579	1.00	32/579	1.00
Used hair dyes	18/978	0.58 (0.30–1.11)	11/415	0.57 (0.28–1.18)	11/615	0.50 (0.24–1.05)
Non-aggressive bladder cancer^i,j^						
Never used hair dyes	36/579	1.00	36/579	1.00	36/579	1.00
Used hair dyes	30/978	0.69 (0.40–1.19)	20/415	0.84 (0.47–1.50)	20/615	0.68 (0.37–1.24)

^a^Permanent dye is defined as a hair color that does not wash out (but grows out), temporary dye as a hair color that washes out over time

^b^Odds ratios were adjusted for age at time of completing the questionnaire, smoking status, smoking duration, and smoking intensity using logistic regression

^c^Never users of any type of hair dye are the referent category for each model

^d^For 112 control participants using both temporary and permanent dyes, for 27 control participants using exclusively temporary dyes, and for 14 participants (1 case and 13 controls) using exclusively permanent dyes, information is lacking on durations of use

^e^For 21 control participants using both temporary and permanent dyes, for 27 control participants using exclusively temporary dyes, and for 14 participants (1 case and 13 controls) using exclusively permanent dyes, information is lacking on number of times per year

^f^For 51 participants (2 cases and 49 controls) using both temporary and permanent dyes, for 52 participants (2 cases and 50 controls) using exclusively temporary dyes, and for 14 participants (1 case and 13 controls) using exclusively permanent dyes, information is lacking on lifetime use

^g^Too few cases for reliable results

^h^The category “aggressive urothelial cell carcinomas” includes TNM stage Tis or T1 and higher or WHO 1973 differentiation grade 3 or WHO/ISUP 2004 high grade

^i^The category “non-aggressive urothelial cell carcinomas” includes TNM stage Ta grade in combination with WHO 1973 differentiation grade 1 or 2 or WHO/ISUP 2004 low grade

^j^39 cases cannot be classified as aggressive or non-aggressive urothelial cell carcinomas, because of lacking information on stage and/or grade

**Table 5:** Risk of bladder cancer stratified by age, educational level and smoking status in women

	Temporary dyes^a^			Permanent dyes^a^		
	Ca/Co	OR (95 % CI)^b^	*p* for interaction	Ca/Co	OR (95 % CI)^b^	*p* for interaction
Age at completion of the questionnaire						
Never used hair dyes^c^	141/1,194	1.00		126/994	1.00	
≤60 years	31/943	0.67 (0.40–1.13)		47/1,190	0.84 (0.46–1.52)	
>60 years	74/450	0.73 (0.52–1.02)	0.90	73/403	0.79 (0.56–1.11)	0.77
Educational level^d^						
Never used hair dyes^c^	141/1,194	1.00		126/994	1.00	
No college degree	71/418	0.79 (0.56–1.12)		80/471	0.76 (0.52–1.11)	
College degree	31/928	0.74 (0.45–1.21)	0.55	35/1,063	0.83 (0.48–1.42)	0.74
Smoking status^e^						
Never used hair dyes^c^	141/1,194	1.00		126/994	1.00	
Never smokers	29/630	1.03 (0.61–1.72)		30/680	1.20 (0.70–2.06)	
Ever smokers	76/763	0.69 (0.49–0.96)	0.20	90/913	0.76 (0.54–1.09)	0.21

^a^Permanent dye is defined as a hair color that does not wash out (but grows out), temporary dye as a hair color that washes out over time

^b^Odds ratios were adjusted for age at time of completing the questionnaire, smoking status, smoking duration, and smoking intensity using logistic regression

^c^Never users of hair dye are the referent category for each model. The reference group for analyses of temporary hair dyes consisted of subjects who had never used temporary hair dyes. The reference group for analyses of permanent hair dyes consisted of subjects who had never used permanent hair dyes

^d^For 47 control participants and 3 cases using temporary dyes, and for 59 control participants and 5 cases using permanent dyes, information is lacking on educational level

^e^Participants who never smoked during their life were defined as never smokers. Former and current smokers were defined as ever smokers

## Discussion

In this case–control study, personal hair dye use was not associated with bladder cancer risk. This finding did not clearly differ by type of hair dye, duration, or frequency of use, dye color, or extent of use.

Our findings are consistent with most previous epidemiological studies [14–24] that have generally reported no association between hair dye use and bladder cancer risk. Similarly, a few meta-analyses have been conducted [28–30]. The interpretation of findings from the meta-analysis by Hunchareck and Kupelnick [30] differs from that of Takkouche et al. [29]. Although both papers evaluated nearly the same set of studies, Hunchareck and Kupelnick [30] suggest an increased bladder cancer risk exists, while Takkouche et al. [29] concludes that there is no association. In the meta-analysis by Hunchareck and Kupelnick sensitivity analyses to examine the influence of hair dye types, color, and study design found the risk of developing bladder cancer increased by 22–50 % among those using permanent hair dye products versus those who do not. The most recent meta-analysis by Kelsh et al. [28] reported associations in individual studies ranging from 0.8 to 1.5, with a meta-relative risk of 1.01 (95 % CI, 0.89–1.14). Thus, the overall results are not yet converging.

In contrast with nearly all other studies, the study of Gago-Dominguez et al. [22] suggested an increased bladder cancer risk of permanent hair dye use (OR, 1.5; 95 % CI, 0.97–2.3), which was more pronounced among females having used hair dyes more than 12 times per year for more than 15 years (OR, 3.3; 95 % CI, 1.3–8.4). Although no overall association between hair dyes and bladder cancer was found in a recently published study from New England, increased risks were observed in certain subgroups [24]. The New England study found that women who used permanent dyes and had at least a college degree had an increased risk of bladder cancer (OR, 3.3; 95 % CI, 1.2–8.9). Our study, however, showed no evidence that the lack of association between hair dyes and bladder cancer risk was modified by educational level. On the other hand, our risk estimates are not robust because of the small number of female cases exposed to hair dyes in some subgroups, resulting in limited statistical power. Also, the New England study found, similar to the California study [31], that the risk was more pronounced among exclusive users of permanent hair dyes who had the NAT2 slow acetylation phenotype (OR, 7.3; 95 % CI, 1.6–32.6). By contrast, a previous study from Spain, the Spanish Bladder Cancer Study, failed to confirm an increased risk of bladder cancer in a population of personal hair dye users and found no increased risk in NAT2 slow acetylators [16]. As data on specific genetic polymorphisms involved in the metabolism of aromatic amines was not available in the current study, we were not able to examine the influence of genetic susceptibility on the risk of bladder cancer associated with hair dye use.

In the present study, we found no gradient in risk with longer duration of hair dye use (i.e., more than 10 years). A consideration with respect to the interpretation of the previous studies is that the numerous chemicals used in hair dyes have varied over time. After the 1980s, several aromatic amines (e.g., 2,4-diaminotoluene, 2,4-diaminoanisole and 4-ABP) were banned as hair dye ingredients after they were found to be carcinogenic in rodents [32]. It is therefore important to examine the time period of dye use because hair dye formulations that occurred around 1980s may be more carcinogenic than newer dyes formulated in response to concern about potential cancer risk. Although we collected detailed information on duration of hair dye use, we have no information about year at first dye use. On the other hand, the presence of the most common permanent hair dye ingredient, *p*-phenylenediamine has remained unchanged for the last 50 years even though animal data support its carcinogenicity [33]. Also, a few new ingredients were introduced during the last 20 years [3]. Bladder cancer has very long latency times, so that effects of historical exposure to aromatic amines in hair dyes may still be observed decades later.

This study may suffer from several limitations inherent to case–control study designs. Information on hair dye use was assessed after the diagnosis of bladder cancer and is therefore sensitive to recall bias. Due to the media attention for this topic, it is possible that cases reported personal hair dye use differently from controls, although it is more likely that this would have resulted in inflated odds ratios. Another limitation of the study is the relatively low response rate among controls, possibly leading to selection bias. A short telephone questionnaire was completed by 100 non-respondent controls to evaluate whether they differed from the participating controls. Compared to non-respondents, respondents were somewhat more highly educated and were more likely to have a paid job (data not shown). Because users of hair dyes among the controls are somewhat higher educated (Table 2), it is possible that we overestimated hair dye use in the general female population that may explain part or all of the observed odds ratios. On the other hand, additional adjustment for educational level did not change the results.

One of the strengths of this study is the information collected about various types of hair dyes (i.e., permanent and temporary dyes). Hair dyes differ in level of suspected carcinogenic chemicals, that is, permanent hair dyes have a higher content of aromatic amines [32]. Nevertheless, we found no substantial difference in risks between types of hair dye. Although we evaluated different types of hair dyes separately, the category of temporary dyes could also be further separated into water-soluble dyes that withstand only one shampooing (temporary dyes) and semi-permanent dyes that are usually synthetic and persist longer than temporary dyes (4–5 shampooings), because these types of products vary slightly in composition. Combining the temporary and semi-permanent dyes may have lead to inability to detect an increased risk for one or the other type. In addition, we stratified the analyses by hair dye color, because dye colors differ in chemical properties, that is, in general dark dyes contain higher concentrations of aromatic amines than lighter shades [32]. A few studies [17, 19] did suggest an increased bladder cancer risk among long-term users of dark dyes. The low prevalence of exposure to dark hair dyes in most populations may explain the discrepancies in these results. We collected information on hair dye color, but were unable to present associations between black dye use and bladder cancer risk because none of the cases reported black hair dye use. We did not find any association for blond, brown, and red hair dye use. It is important to note, however, that these findings were also based on small numbers of cases.

## Conclusion

We did not confirm an increased risk of bladder cancer with use of hair dyes in this population. We observed no association between temporary or permanent hair dye use and bladder cancer risk among women. The lack of association was not affected by duration or frequency of use, dye color, or extent of use. Because hair dye use is most frequently used by women while bladder cancer is more prevalent among men, it is difficult to study the role of hair dye. In our analyses, the number of female cases is fairly small which affects the power of the study to detect small increases in risk. Residual confounding by smoking or selection bias can also not entirely be excluded. Further investigations with larger sample sizes, focussing on subgroups that may be more prone to the toxic effects of hair dyes, may be needed to obtain a definitive answer to the question. However, because both the risk and the etiological fraction induced by hair dyes are probably small, it may be better to focus on yet unidentified bladder cancer factors.
